# Medicinal Plants Traditionally Used to Treat Digestive System Disorders in Lithuania

**DOI:** 10.3390/plants15091390

**Published:** 2026-04-30

**Authors:** Birutė Karpavičienė

**Affiliations:** Laboratory of Economic Botany, State Scientific Research Institute Nature Research Centre, Akademijos Str. 2, 08412 Vilnius, Lithuania; birute.karpaviciene@gamtc.lt

**Keywords:** ethnobotany, herbal medicine, diarrhea, constipation, appetite stimulant

## Abstract

The popularity of herbal remedies is on the rise, but this often comes at the expense of general knowledge about non-native species. The frequency and versatility of the use of medicinal plants does not depend on their origin, while the use of species with proven efficacy is much more intensive. The most abundant plant families, according used taxa and use records, are Asteraceae, Rosaceae and Lamiaceae. The greatest consensus is on the choice of taxa suitable for the treatment of bloating/flatulence and diarrhea, most commonly treated with *Carum carvi* L. fruit tea and dried or fresh berries of *Vaccinium myrtillus* L., respectively. The most popular species for treatment of digestive disorders are *Artemisia absinthium* L. and *Matricaria chamomilla* L. The use of different taxa for the treatment of digestive disorders in Lithuania varies considerably for a number of reasons, one of which is the uneven distribution of medicinal plant species in the study areas.

## 1. Introduction

For centuries, people have turned to traditional healers, home remedies and ancient medical knowledge to address their health and well-being needs [1]. Traditional medicines are not only incorporated into people’s daily healthcare practices, but also hold symbolic significance in terms of national identity [2]. The study of the interactions between plants and people, including the influence of plants on human culture, is the focus of the interdisciplinary field of ethnobotany [3]. It is a multidisciplinary science that collaborates with many other disciplines, including chemistry and pharmacology [4]. In most cases ethnopharmacology can be considered as a branch of ethnobotany [5]. It draws on methodologies and methods from various disciplines, and various ethnobotanical indices are often used to transform primary data into statistical measures [6]. Ethnobotanical information and knowledge are believed to have contributed to the development of close to 30% of modern medicines [7]. Therefore, the identification, documentation, transmission, revitalization and promotion of traditional knowledge is essential to ensure its preservation and vitality [8].

However, the use of traditional medicines was challenged by the advent of semi-synthetic and synthetic drugs since the nineteenth century, and more so in the twentieth century [9]. Nevertheless, the use of herbal medicines has increased considerably over the last decades worldwide, as many people now turn to these products for a wide range of health problems [10]. Similarly, the use of medicinal plants is increasing in Lithuania, mainly due to preserved or revived traditions and the preference for natural products [11]. On the other hand, the popularity of certain natural remedies for human health has led to the popularization of common knowledge about some widely marketed, often non-native species, thus promoting the substitution of traditionally used native ones [12]. As a result, traditional knowledge is being lost with the increasing use of introduced plants [13]. Prakofjewa et al. [14], who studied traditional knowledge in rural settlements in the Vilnius region (Lithuania), found that a very large proportion (approximately 80%) of reported uses were associated with wild plant species. However, there is a fundamental difference between “wild” and “native,” as a number of naturalized alien medicinal plant species can be found in the wild [15,16]. There is no study examining the differences between the use of native and non-native plant species in traditional Lithuanian medicine.

In traditional medicine, plants are most often used to treat milder disorders, especially those of the digestive, dermal and respiratory systems [17,18,19,20]. Moreover, phytotherapy is also an important part of the treatment of these diseases in modern medicine [21,22,23]. Accordingly, among the herbal medicinal products listed in the Register of Medicinal Products of the Republic of Lithuania, the most common are those affecting the digestive system [24]. In the European Union, a common regulatory framework applies to all Member States, and harmonized quality standards for medicinal products are published in the European Pharmacopeia after adoption by the European Pharmacopeia Commission [25]. Meanwhile, the European Medicines Agency (EMA) is a decentralized agency of EU responsible for the scientific evaluation, supervision, and safety monitoring of medicines in the EU [26].

Unlike in Western European countries, attitudes toward traditional medicine in Lithuania were affected by the Soviet era. In Soviet Lithuania, traditional medicine was regarded as a historical relic, and according to researchers of the time, folk medicine was in decline; the data collected was considered to have only cognitive and historical significance [27]. Until the 21st century, ethnobotanical research in Lithuania was dominated by the ethnologist’s view of folk medicine, where the only thing that mattered was which herb cured which disorder [28]. Similarly, in all countries of Northeast Europe, data on the use of plants and people’s attitudes toward them have been studied primarily within the fields of the humanities (e.g., ethnology and folklore studies) [29]. In the last two decades, when pharmacy students at the Lithuanian University of Health Sciences began to choose ethnopharmacological research as the basis for their master’s thesis, research on traditional medicine in Lithuania has intensified [30]. The works published to date have been limited to ethnobotanical studies in small areas, involving a relatively small number of informants, and usually remain unpublished.

The aim of this study is to review and analyze ethnobotanical and ethnopharmacological research data related to the traditional use of plants and to identify trends in the use of plants for the treatment of digestive disorders in Lithuania.

## 2. Results

The number of respondents vary from 9 to 67 per study, with a total of 476 respond-ents, of which only 56 are men. There are no special studies on the differences between men and women’s knowledge about medicinal plant use in Lithuania, but according to Prakofjewa et al. [14], 50 women and 15 men reported on an average of 14.9 and 9.9 plant taxa, respectively, and this difference is statistically significant.

### 2.1. Influence of Plant Origin on Its Use

A total of 341 taxa are recorded, of which more than half, i.e., 184 plant taxa and one lichen species, are used to treat digestive disorders. In the studies reviewed, between 15.3% and 58% (an average of 37.1%) of the recorded taxa are used to treat digestive system disorders, while the relative proportion of use records for digestive system disorders ranges from 11.9% to 29.8% and averages 20.5%. Of the 185 taxa, 102 taxa are native and 83 non-native. Native plant taxa account for 46.9–87.9% (66.4% on average) in the reviewed studies, with the exception of Bajoraitė [31], who focused more on cultivated plants. Studies on medicinal plants used by ethnic minorities living in Lithuania [32,33,34] do not differ in this respect (67.4, 75.8, 64.4% of native taxa, respectively). The results of a study by Prakofjewa et al. [14], who investigated the plants used by Lithuanians and Poles living in the same area, show that respondents from both ethnic groups cited the same proportion of native plant species for treating digestive disorders (76.3% and 76.5%, respectively). Although native taxa are used slightly more frequently than non-native taxa, there are no statistically significant differences between them in the number of studies in which the taxon are recorded, the number of use records and the number of disorder groups (Table 1). Similarly, when comparing the use values (UV) of native and non-native plant taxa, no statistically significant difference was found in any of the five detailed studies (*p* > 0.05, Mann–Whitney U test). No statistically significant difference was found even in the study by Prakofjewa et al. [14] (Table 2), which reveals that approximately 80% of UV involved wild plant species. This can be explained only by the high proportion of native species (72.5%) recorded in the study.

### 2.2. Influence of Plant Efficiency on Its Use

A total of 70 species of plants and one species of lichen are registered in EMA mono-graphs for the treatment of digestive disorders [26]. Of these, 35 plant taxa and one lichen species were registered in this study, representing 19.5% of the total number of registered taxa. All characteristics differed statistically between EMA-approved and EMA non-approved taxa groups (Table 1). EMA-approved species are much more popular than EMA non-approved taxa. When the taxa were divided into four groups according to their origin and EMA approval (Table 3), it was found that regardless of origin, EMA-approved taxa are used significantly more often. However, there are exceptions, e.g., *Plantago lanceolata* L., one of the three species of the genus widespread in Lithuania, approved by the EMA as a demulcent for the symptomatic treatment of oral irritation, which is mentioned in only one study (4.8%), while non-approved *Plantago major* L. is registered in 13 studies (61.9%).

### 2.3. Taxonomical Diversity

The registered plants belong to 63 families. The most abundant family is Asteraceae, which leads not only in the number of taxa (24) but also in the number of use records (293) and the proportion of EMA-approved species (37.5%). The next most abundant families are Rosaceae, Lamiaceae and Apiaceae (Table 4). However, in the Lamiaceae and Apiace-ae a large number of non-native taxa are used, while in the Ericaceae and Plantaginaceae families only native species are registered (Table 4). In general, Asteraceae, Rosaceae and Lamiaceae are the most commonly used families in all the studies reviewed. Among the recorded species, two plant species, namely *Arnica montana* L. (recorded in two studies), and *Gentiana cruciata* L. (one study) are rare and included in the Red Data Book of Lithuania [37].

### 2.4. Plant Use According Digestive Disorders Groups

The greatest consensus is on the choice of taxa suitable for the treatment of bloat-ing/flatulence and diarrhea (Fic = 0.64 and 0.60, respectively, Table 5). Bloating or flatulence is usually treated with *Carum carvi* L. fruit decoction (48% of studies). More often, this tea is used as a digestive (62% of studies). In some regions of Lithuania, *Carum carvi* decoction is usually used instead of regular tea [30]. Diarrhea is most commonly treated with dried or fresh berries of *Vaccinium myrtillus* L., tea of *Artemisia absinthium* L. and decoction or tincture of *Quercus robur* L. bark (registered in 76, 62 and 57% of studies, respectively). In addition to these species used to treat diarrhea, the most commonly agreed uses were *Carum carvi* for digestive problems (62%), *Matricaria chamomilla* for relieving of pain and spasms (52%), *Calendula officinalis* L. as a stomatic (48%) and *Allium sativum* L. as an anthelmintic (48%).

### 2.5. Traditional and Approved Uses of Plants

The use of two plant species, *Artemisia absinthium* and *Matricaria chamomilla* L., was mentioned in all of the studies reviewed. *Matricaria chamomilla* is used to treat almost all categories of ailments related to the digestive system, with the exception of ulcers and helminthiasis. It is most commonly used for pain and spasms, as a stomatic, carminative and digestive (Table 6). The European Union Herbal Monograph refers to almost the same therapeutic areas, while *Artemisia absinthium* herb preparations can be used for temporary loss of appetite or for mild heartburn and stomach/gut disorders [26]. Of the ten most popular species used to treat digestive disorders, only one species, *Allium sativum*, does not have EMA-approved indications. Furthermore, the main indications for the seven most popular species coincide with those listed in the EMA monographs [26].

### 2.6. Variation Between Localities

Due to a variety of reasons, one of which is the uneven distribution of the medicinal plant species in the study areas, the use of different taxa to treat digestive disorders in Lithuania varies widely. However, this cannot explain the differences in the use of the most popular species, which are common native or cultivated plants. The results of the five most comprehensive studies (Table 7) show that UV of even the most popular species varies considerably from one location to another. For example, the use of *Artemisia absinthium* for various digestive disorders was mentioned between 0.18 and 1.19 times per respondent.

## 3. Discussion

In the studies reviewed, the majority of respondents were women. This is because herbal medicine is a woman’s occupation in Lithuania [38]. Although men are also knowledgeable about herbs and use them in a veterinary context, herbal medicine is considered ‘not a man’s business’ [39]. Similarly, in Brazil and Northern Ecuador, women, especially older women, are the main providers of family and community health services [40,41]. Women’s double role of family food and care providers may explain their expertise in medicinal plant use [42].

The diversity of plants used in traditional medicine depends on cognitive characteristics, ecological factors and cultural history [43]. Ecological factors include the composition of the local flora and the availability of medicinal plant species. The most species-rich plant families in Lithuanian flora are Asteraceae, Poaceae, Cyperaceae and Rosaceae [44]. The largest number of native species used for treatment of digestive disorders belong to the families Asteraceae and Rosaceae, while only two native species of the Poaceae are recorded with five UR, and no plants belonging to the family Cyperaceae are used at all. The chemical compounds with medicinal value are not equally distributed among different botanical families and the underuse of Poaceae and Cyperaceae families can be explained by their low biological activity [45].

According to Zenderland et al. [46], the proportion of wild species among plants used for medicinal purposes in 19 studies varies from 28.2% to 90.9%, and use value is higher for cultivated plants than for wild plants. In Lithuania, native species slightly outnumber non-native species. It could be speculated that inexperienced researchers may have missed some of the less common and more difficult to identify native species, as only a few studies have mentioned unidentified plants. However, in a study conducted by a botanist [30], the proportion of native species was similar (64.9%).

Digestive diseases may be treated with a wide range of bioactive compounds, which might explain the large number of plants used for this purpose [47]. For functional gastrointestinal disorders, plants with a predominance of bitter substances that stimulate gastric secretion and gastrointestinal motility, essential oils with spasmolytic activity or with peristalsis-stimulating effects, spasmolytic alkaloids, and other constituents are used [23]. The main biologically active compounds in most of the popular species, namely *Artemisia absinthium*, *Matricaria chamomilla*, *Carum carvi*, *Mentha* spp., *Achillea millefolium*, and *Melissa officinalis*, are found in essential oils [48,49,50,51,52,53], and the EMA-approved indications for use of these plants are minor gastrointestinal disorders, including bloating and flatulence [26]. *Artemisia absinthium* and *Achillea millefolium* also contain biologically active compounds that provide bitterness, e.g., sesquiterpene lactones (absinthin and achillin) and volatile compounds (thujone and camphor), which stimulate the appetite and improve digestion [53,54,55,56]. The main active compounds in *Calendula officinalis* flowers are terpenoids with extensive anti-inflammatory activity and flavonoids, particularly quercetin, which have significant wound-healing properties [57]. The main biologically active compounds of *Vaccinium myrtillus* berries are classified as a tannin–anthocyanin complex [58]. The use of *Vaccinium myrtillus* berries to treat diarrhea is the most widely recognized in Lithuania and has been confirmed by as many as 76% of the studies reviewed. A decoction or alcoholic tincture of *Quercus robur* bark is also mentioned in many studies as a remedy for diarrhea. Because of its high tannin content, oak bark possesses astringent, desiccating, emplastic, antimicrobial and anti-inflammatory activities [59]. However, it is noted that this remedy is more suitable for veterinary use [30].

The most popular used plant species are common native or frequently cultivated species in Lithuania. Sometimes these groups overlap. *Artemisia absinthium* is found in poor soils, so its distribution in the country is very uneven, which is why it is often cultivated. *Matricaria chamomilla* used to grow in fields in Lithuania as a weed, but it is now rarely found in the wild and is only cultivated [30], like *Calendula officinalis*, which is traditionally grown as an undemanding annual ornamental and medicinal plant. The distribution of plant species can be important for differences in plant use between communities [47]. However, historical and cultural factors also play a significant role. For example, *Chenopodium album* L. is one of the six most popular species used to treat digestive disorders in Nepal [60], and *Trifolium repens* L. is the second most popular species used for this purpose in northern Pakistan [61], while there is not a single record of the use of these widespread species in Lithuania to treat gastrointestinal disorders. The results of our study also confirm that, according to De Medeiros and Albuquerque [47], the environment plays an important role in the selection of medicinal plants and individuals from different ethnic groups or origins inhabiting nearby or neighboring regions in similar environments tend to use similar repertoires of medicinal plants.

According to Juodelytė [62], 17% of the respondents use medicinal plants, with *Mat-ricaria chamomilla*, *Carum carvi*, *Artemisia absinthium*, *Mentha* spp. and *Calendula officinalis* being the most popular. The results of the above-mentioned survey, conducted in one Lithuanian city, are in line with the results of this study. Some medicinal plants, such as *Ruta graveolens*, *Calendula officinalis*, *Tanacetum balsamita* L., *Mentha* spp. and *Tropaeolum majus* L., have been cultivated in Lithuania for a long time, together with ornamental plants [63,64], while others, e.g., *Perilla frutescens* (L.) Britton, were introduced only at the end of the last century [65]. On the other hand, there are some plant species that are no longer used, or are used very rarely. Some of them are poisonous, such as *Daphne mezereum* L. and *Datura stramonium* L., used for dental treatment. Some plants, e.g., *Bryonia alba* L., *Lysimachia nummularia* L., *Peucedanum palustre* (L.) Moench and *Thalictrum lucidum* L., go into oblivion along with the methods of preparation and the disorders they treat [30]. One of such disorders is a rupture, the folk name for abdominal pain and loss of appetite caused by overwork or the lifting of too heavy a weight. According to Damskytė [66], a rupture seems to be the term for all abdominal diseases that cause sharp pains without diarrhea.

## 4. Materials and Methods

### 4.1. Study Area

Lithuania is located in the Baltic Sea region, in the temperate zone, and covers an area of 65,300 km^2^. The country is mostly flat, except for some hilly areas. The highest altitude is 294 m above sea level. The terrain is rich in lakes and wetlands and more than 33% of the country is forested. The native flora of Lithuania includes about 1350 species of vascular plants, of which 457 are considered medicinal [67,68].

### 4.2. Data Collection

**Figure 1:**
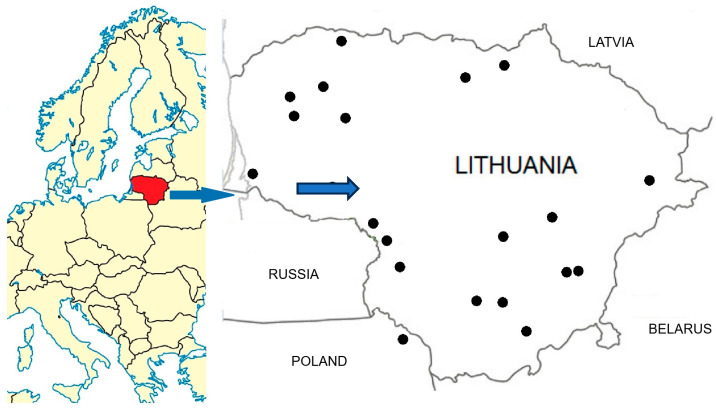
Locations in Lithuania of the studies reviewed (Table 8).

Published studies in online databases (PubMed) and final theses of the Lithuanian University of Health Sciences (LSMU) in institutional repository (https://lsmu.lt/biblioteka/ (accessed on 16 February 2026)) covering the period from 2000 to 2026 were checked for comprehensive ethnobotanical and ethnopharmacological studies in Lithuania. Original studies that specified the study location, the number of respondents, a complete list of plants, and their indications for use were selected. A PubMed search for “medicinal plants” and “Lithuania” yielded 94 results; adding “digestive” narrowed the results to eight, of which only four met the criteria for original research. A search of the LSMU institutional repository using the keywords ‘ethnopharmaceutical’ or ‘ethnobotanical’ in Lithuanian yielded 26 theses, eight of which either lacked a complete list of plants or were not related to digestive system disorders and two theses served as the basis for published papers. A total of five published original studies and 16 master’s theses were selected, examining the traditional use of medicinal plants to treat digestive disorders in various regions of Lithuania (Figure 1, Table 8). Among them, four studies investigated traditional medicinal knowledge of ethnic minorities [14,32,33,34]. Only five of the selected studies provided comprehensive information on the plant species listed, their medicinal uses, parts, preparation methods, and detailed usage reports [14,30,31,35,36].

The digestive disorders mentioned in the studies were grouped into 15 groups (DG): digestive problems, diarrhea, constipation, pain and spasms, stomach problems, liver problems, mouth and teeth, lack of appetite, inflammation, acidity, gallbladder problems, gastric ulcer, helminths, and non-specified problems. It was not always clear which group to assign the disorder to, so the grouping is quite approximate.

The accuracy of the species names given in this document depends on the original sources, with some modifications. As most of the studies reviewed are by student pharmacists, the accuracy of identification of the more difficult to identify species is highly questionable. According to Łuczaj [82], in ethnobotanical studies, the most common species identification errors occur when an informant refers to a plant species by a local name that is identical or similar to the official scientific name of the species, and the plant is identified under the assumption that the local name refers to the same taxon. However, it is not possible to verify the identification because the voucher specimens were not made or were made from a plant collected by the author without the informant’s approval. Some apparently misidentified species were not included in the study. Several genera with more than one species occurring in Lithuania and that are difficult for non-botanists to identify, such as *Alchemilla* L., *Aloe* L., *Arctium* L., *Betula* L., *Crataegus* L., *Dryopteris* Adans., *Mentha* L., *Rosa* L., *Rumex* L., excluding *Rumex acetosa*, and *Thymus* L., were described at the level of genera. Therefore, the term taxon will be used hereafter to refer to plants identified at different levels (species and genus).

### 4.3. Data Analysis

All recorded taxa were grouped according to their occurrence in Lithuania: native (wild) and non-native (cultivated or purchased). The boundaries between wild and cultivated taxa are not very clear and the distinction is based on frequency. Some of the less common or often used native species, such as *Artemisia absinthium*, *Origanum vulgare*, *Humulus lupulus*, *Rubus idaeus* are sometimes cultivated. Nevertheless, such species were included in the native group. Conversely, *Matricaria chamomilla* used to be a common weed in fields, but now it is only cultivated and has been classified as non-native.

A further grouping was made on the basis of whether the taxon is included in the European Union Herbal Monograph published by the European Medicines Agency (EMA) as suitable for the treatment of gastrointestinal disorders [26]. The following therapeutic areas were covered: gastrointestinal disorders, loss of appetite, constipation, digestive disorders ant partial mouth and throat disorders (inflammations in the mouth of the oral mucosa). A group comprising species included in the European Pharmacopeia is referred to as EMA-approved and a group comprising species not included in the European Pharmacopeia is referred to as EMA non-approved.

The use record (UR) refers to the use of a particular taxon for the treatment of a specific group of disorders. This means that if the same taxon is mentioned in the same study to treat different groups of disorders, each of them is considered as a different use record. Only six studies (three of them by the author) provided use records for each taxon for a certain application. The data of these studies were analyzed to assess differences in the use of individual taxa in different locations across Lithuania and the use values (UV) for each taxon were calculated as follows:

UV = ΣUR/n,(1)

where UR = use record, n = total number of respondents.

To test whether the DG for each taxon in the reviewed studies could at least partially replace the UR, a correlation was calculated between the UR and DG for each of the taxa in each of the six studies. A significant correlation between these indices was found (r = 0.74, *p* < 0.001, n = 245), so a modified use records (UR) related to the studies rather than the respondents was used to assess the intensity of use of plant taxa.

The consensus factor (Fic) was calculated according to Heinrich et al. [83] as follows:

Fic = (UR − NT)/(UR − 1)(2)

where UR = use records in each DG, and NT = number of taxa used in each DG. A high Fic value (close to 1) indicates that a large proportion of the studies registered relatively few taxa, while a low value indicates that different taxa are used for a given group of disorders in different locations (studies).

Statistical analysis of the data was performed using the Statistica 10.0 software package (StatSoft Inc., Hamburg, Germany). The non-parametric Mann–Whitney U test was used to determine differences between two independent groups, e.g., taxa origin and EMA approval, while significant differences between multiple groups were tested using the Kruskal–Wallis test.

## 5. Conclusions

In Lithuania, more than half of all plant taxa traditionally used as medicinal are used to treat digestive disorders. The frequency and versatility of medicinal plant use is independent of their origin, while the use of species with proven efficacy is much more intense. Although only 36 of the 185 species used to treat digestive disorders are EMA-approved, as many as nine of them are in the top ten. More than half of the studies support the use of *Vaccinium myrtillus*, *Artemisia absinthium* and *Quercus robur* as an anti-diarrheal, *Carum carvi* as a digestive, and *Matricaria chamomilla* as a remedy for pain and spasms. The European Union Herbal Monograph lists almost the same therapeutic areas for these species. Local species distribution also plays an important role in medicinal plant use.

## Figures and Tables

**Table 1:** Descriptive statistics (mean ± SD) and *p*-value from Mann–Whitney U test of medicinal plant taxa grouped by its origin and approval by European Medicines Agency [26], based on a review of 21 studies. NS = the number of studies in which the taxon are recorded; UR = the number of use records; DG = number of disorder groups.

Characters	Native (n = 102)	Non-Native (n = 83)	*p* Value	Approved (n = 36)	Non-Approved (n = 149)	*p* Value
NS	4.4 ± 4.7	3.8 ± 4.3	0.536	8.8 ± 6.5	3.0 ± 3.0	<0.001
UR	7.1 ± 10.4	6.1 ± 10.1	0.371	17.3 ± 17.4	4.1 ± 5.1	<0.001
DG	3.7 ± 3.1	3.1 ± 2.9	0.153	6.2 ± 3.9	2.7 ± 2.3	<0.001

**Table 2:** The use values (mean ± SD) and *p*-value from Mann–Whitney U test of medicinal plant taxa grouped by its origin in the five most comprehensive surveys. n = number of taxa.

Origin	[14] n = 40	[30] n = 43	[31] n = 48	[35] n = 53	[36] n = 48
Native	0.09 ± 0.21	0.12 ± 0.16	0.19 ± 0.24	0.14 ± 0.11	0.18 ± 0.33
Non-native	0.06 ± 0.05	0.10 ± 0.15	0.15 ± 0.21	0.14 ± 0.09	0.17 ± 0.20
*p* value	0.889	0.308	0.793	0.963	0.631

**Table 3:** Descriptive statistics (mean ± SD) on the characteristics of plant taxa used in Lithuania for the treatment of gastrointestinal disorders, and grouped according to origin and approval of European Medicines Agency [26], based on a review of 21 studies. NS = number of studies in which the taxon are recorded; UR = number of use records; DG = number of disorder groups.

Groups	N	NS	UR	DG
Native approved	21	9.8 ± 5.9 a ^1^	18.4 ± 16.0 a	7.0 ± 3.3 a
Native non-approved	81	3.0 ± 3.0 b	4.2 ± 5.6 b	2.8 ± 2.5 b
Non-native approved	15	7.5 ± 7.2 ab	15.7 ± 19.6 ab	5.3 ± 4.6 ab
Non-native non-approved	68	3.0 ± 2.9 b	4.0 ± 4.5 b	2.6 ± 2.1 b

^1^ Values followed by the different letters differ significantly between groups according to the Kruskal–Wallis test (*p* ≤ 0.005).

**Table 4:** The most abundant plant families used to treat digestive system disorders in Lithuania, based on a review of 21 studies. EMA-approved = relative proportion of taxa approved by European Medicines Agency [26]; UR = the number of use records.

Family	Taxa Number (Native)	EMA Approved, %	UR
Asteraceae	24 (13)	37.5	293
Rosaceae	20 (12)	25.0	96
Lamiaceae	15 (5)	33.3	126
Apiaceae	12 (4)	8.3	114
Ericaceae	7 (7)	14.3	54
Brassicaceae	7 (2)	0	34
Fabaceae	6 (2)	16.7	8
Polygonaceae	7 (4)	14.3	38
Poaceae	5 (2)	0	10
Plantaginaceae	5 (5)	0	38

**Table 5:** Medicinal plant taxa number (NT), use records (UR), consensus factor (Fic) and the most popular taxa according to disorders categories indicating the percentage (PS) of studies in which they are mentioned, from the 21 studies reviewed.

Category of Disorders	NT	UR	Fic	The Most Popular Taxa with PS
Digestive problems	73	171	0.58	*Carum carvi* 62, *Artemisia absinthium* 43, *Mentha* spp. 43, *Matricaria chamomilla* 33, *Origanum vulgare* 33, *Melissa officinalis* 29, *Calendula officinalis* 29
Diarrhea	52	129	0.60	*Vaccinium myrtillus* 76, *Artemisia absinthium* 62, *Quercus robur* 57, *Carum carvi* 33, *Rumex* spp. 29, *Plantago major* 29
Pain, spasms	49	89	0.45	*Matricaria chamomilla* 52, *Artemisia absinthium* 38, *Mentha* spp. 29
Stomach problems	45	75	0.41	*Artemisia absinthium* 24, *Symphytum officinale* 24
Liver problems	49	82	0.41	*Helichrysum arenarium* 29, *Calendula officinalis* 29, *Taraxacum officinale* 24
Mouth, teeth	45	98	0.55	*Calendula officinalis* 48, *Matricaria chamomilla* 38, *Quercus robur* 33, *Allium sativum* 29, *Salvia officinalis* 29
Constipation	42	80	0.48	*Sorbus aucuparia* 38, *Frangula alnus* 29, *Rheum rhabarbarum* 29, *Linum usitatissimum* 24
Los of appetite	48	89	0.47	*Artemisia absinthium* 38, *Menyanthes trifoliata*33, *Acorus calamus* 29
Inflammation	39	66	0.42	*Matricaria chamomilla* 29, *Calendula officinalis* 24, *Plantago major* 24
Acidity	36	51	0.30	*Solanum tuberosum* 24, *Vaccinium oxycoccos* 19
Gallbladder problems	28	36	0.23	*Helichrysum arenarium* 14, *Mentha* spp. 14, *Taraxacum officinale* 14
Ulcer	27	55	0.52	*Solanum tuberosum* 24, *Aloe* spp. 24
Helminthiasis	21	50	0.59	*Allium sativum* 48, *Artemisia absinthium* 43, *Tanacetum vulgare* 33
Bloating, flatulence	17	45	0.64	*Carum carvi* 48, *Matricaria chamomilla* 38, *Mentha* spp. 24, *Anethum graveolens* 24
Not specified	64	120	0.47	*Artemisia absinthium* 33, *Matricaria chamomilla* 29, *Hypericum perforatum* 29, *Plantago major* 24

**Table 6:** Plant taxa registered at least in five of the 21 studies reviewed, the main disorders categories with number of study that mentioned it and EMA indications related to digestive system disorders. NS = number of studies in which the taxon are recorded; UR = number of use records; DG = number of disorder groups. The main disorders corresponding to the EMA indications are in bold.

Taxa	NS	UR	DG	Main Disorders, NS	EMA Indications [26]
*Artemisia absinthium* L.	21	65	12	Diarrhea 13, **digestive 9**, anthelmintic 9, **appetite stimulant 8**, pain and spasms 8	Temporary loss of appetite; mild dyspeptic/gastrointestinal disorders
*Matricaria chamomilla* L.	21	58	13	**Pain and spasms 11, carminative 8**, **stomatic 8**, digestive 7	Minor gastro-intestinal complaints such as bloating, spasms; minor ulcers and inflammations of the mouth
*Calendula officinalis* L.	19	45	11	**Stomatic 10**, digestive 6, liver 6, inflammation 5,	Minor inflammations in the mouth or the throat
*Carum carvi* L.	19	49	12	Digestive 13, **carminative 10**, diarrhea 7	Digestive disorders such as bloating and flatulence
*Vaccinium myrtillus* L.	19	33	9	**Diarrhea** 16	Mild diarrhea; minor inflammations of the oral mucosa
*Mentha* L.	17	49	14	**Digestive 9**, pain, spasms 6, **carminative 5**	*M. piperita*: digestive disorders such as dyspepsia and flatulence
*Quercus robur* L.	17	27	8	**Diarrhea 12, stomatic 7**	Mild diarrhea; minor inflammation of the oral mucosa
*Achillea millefolium* L.	14	22	12	Inflammation 4, digestive 3	Temporary loss of appetite; mild, spasmodic gastrointestinal complaints including bloating, and flatulence
*Allium sativum* L.	14	24	7	Anthelmintic 10, stomatic 6	–
*Melissa officinalis* L.	14	22	8	Digestive 6	Mild gastrointestinal complaints including bloating and flatulence
*Sorbus aucuparia* L.	14	19	10	Laxative 8	–
*Acorus calamus* L.	13	27	11	Appetite stimulant 6, stomach 4	–
*Hypericum perforatum* L.	13	30	12	Not specified 6, liver 4, stomach 4	Mild gastrointestinal discomfort
*Menyanthes trifoliata* L.	13	26	11	**Appetite stimulant** 7, **digestive** 4	Temporary loss of appetite; mild digestive disorders such as bloating and flatulence
*Plantago major* L.	13	33	11	Diarrhea 6, inflammation 5	–
*Helichrysum arenarium* (L.) Moench	12	20	7	Liver 6	Digestive disorders with a feeling of fullness, bloating
*Symphytum officinale* L.	12	18	8	Stomach 5	–
*Taraxacum officinale* F.H.Wigg.	12	20	9	Liver 5	Mild digestive disorders such as feeling of abdominal fullness, flatulence, and slow digestion; temporary loss of appetite
*Daucus carota* L.	11	14	8	Diarrhea 3, laxative 3	–
*Linum usitatissimum* L.	11	22	8	Laxative 5, inflammation 4	Mild gastrointestinal discomfort
*Allium cepa* L.	10	14	7	Appetite stimulant 3	–
*Anethum graveolens* L.	10	18	9	Carminative 5	–
*Potentilla erecta* (L.) Raeusch.	10	17	6	**Diarrhea** 5; **stomatic** 4	Mild diarrhea; inflammations of the oral mucosa
*Solanum tuberosum* L.	10	14	5	Acidity 5, ulcer 5	–
*Armoracia rusticana* G.Gaertn., B.Mey. & Scherb.	9	14	8	Appetite stimulant 4	–
*Origanum vulgare* L.	9	13	6	Digestive 7	–
*Salvia officinalis* L.	9	12	6	**Stomatic** 6	Mild dyspeptic complaints such as heartburn, bloating; inflammations in the mouth
*Arctium* L.	8	10	6	Digestive 3	*A. lappa*: temporary loss of appetite
*Fragaria vesca* L.	8	11	5	Liver 4	Mild diarrhea
*Frangula alnus* Mill.	8	9	4	**Laxative** 6	Occasional constipation
*Petroselinum crispum* (Mill.) Fuss	8	10	6	Digestive 3	*–*
*Tanacetum vulgare* L.	8	12	6	Anthelmintic 7	–
*Urtica dioica* L.	8	10	9	Ulcer 2	–
*Aloe* L.	7	11	6	Ulcer 4	Occasional constipation
*Centaurium erythraea* Rafn	7	12	6	**Digestive** 4	Mild dyspeptic/gastrointestinal disorders; temporary loss of appetite
*Hippophae rhamnoides* L.	7	7	3	Ulcer 4	–
*Humulus lupulus* L.	7	9	5	Digestive 3	–
*Rumex* L.	7	12	5	Diarrhea 6	–
*Vaccinium oxycoccos* L.	7	8	5	Acidity 4	–
*Ocimum basilicum* L.	6	7	3	Digestive 5	–
*Rheum rhabarbarum* L.	6	8	3	Laxative 6	–
*Betula* L.	5	10	9	Acidity 2	–
*Persicaria bistorta* Samp.	5	7	5	Stomatic 2	–
*Brassica oleracea* L.	5	6	5	Ulcer 2	–
*Capsella bursa-pastoris* (L.) Medik.	5	6	4	Diarrhea 3	
*Centaurea cyanus* L.	5	5	4	Liver 2	–
*Chelidonium majus* L.	5	7	4	Liver 3	–
*Epilobium angustifolium* L.	5	8	4	Not specified 3	–
*Polygonum aviculare* L.	5	7	4	Liver 3	Minor inflammations in the mouth
*Rosa* L.	5	5	4	Digestive 2	Mild inflammations of the oral mucosa
*Tussilago farfara* L.	5	5	4	Liver 2	–
*Viburnum opulus* L.	5	6	5	Stomatic 2	–

**Table 7:** The top seven plant taxa, registered at least in 80% of the 21 studies reviewed with use values (UV) in the five most comprehensive surveys. n = number of recipients.

Taxon	[14] n = 67	[30] n = 30	[31] n = 27	[35] n = 28	[36] n = 30
*Artemisia absinthium*	1.19	0.80	0.78	0.18	1.10
*Matricaria chamomilla*	0.13	0.63	0.33	0.29	0.53
*Calendula officinalis*	0.13		0.33	0.18	0.63
*Carum carvi*	0.16	0.20	0.52	0.36	0.87
*Vaccinium myrtillus*	0.33	0.23	0.07	0.57	
*Mentha* spp.	0.15	0.03	1.19	0.32	0.27
*Quercus robur*	0.03	0.17		0.32	0.13

**Table 8:** Data of ethnopharmacological and ethnobotanical studies reviewed in the research. DD = number of taxa used to treat digestive system disorders; DD UR = relative proportion of use records for digestive system disorders.

Study	No. of Recipients, Women/Men	Number of Taxa		DD UR, %
		Total	DD (Native)	
Aleknavičienė [69]	11/0	101	32 (15)	29.4
Bajoraitė [31]	21/6	97	48 (17)	18.0
Bieliauskaitė [32]	8/4	110	33 (29)	17.9
Ivaškevičienė [70]	23	65	19 (9)	29.8
Karpavičienė [30]	29/1	103	43 (29)	21.5
Kvederaitė [71]	18/2	99	41 (23)	20.0
Kvederavičiūtė [33]	14/2	78	45 (29)	23.0
Mafanova [34]	10/2	111	43 (29)	18.0
Mališauskaitė [72]	9/1	77	14 (11)	11.9
Narkutė [73]	22	51	15 (11)	17.1
Los [74]	13/2	170	56 (37)	–
Petkevičiūtė et al. [75]	19/1	79	25 (18)	27.0
Prakofjewa et al. [14]	50/17	139	40 (29)	–
Pranskūnienė et al. [35]	24/4	125	53 (34)	19.3
Ratkevičiūtė [36]	29/1	120	48 (29)	21.1
Puidokaitytė [76]	24/0	69	40 (26)	–
Staugaitis [77]	8/1	46	20 (16)	18.0
Ščerba [78]	33/3	103	25 (14)	16.1
Šimkutė [79]	17/3	111	17 (14)	–
Vinslauskaitė [80]	29/5	79	31 (22)	20.2
Woznalis [81]	9/1	117	44 (22)	20.0
Total	420/56	341	185 (102)	

## Data Availability

These data were derived from the following resources available in the public domain: [https://lsmu.lt/biblioteka/] (accessed on 20 January 2026).

## Abbreviations

The following abbreviations are used in this manuscript:

DD	The number of taxa used to treat digestive system disorders
DG	The number of disorder groups
EMA	European Medicines Agency
Fic	Consensus factor
n	Sample size
NS	The number of studies in which the taxon are recorded
NT	The number of taxa
PS	Percentage of studies
SD	Standard deviation
SN	The number of studies in which the taxon are recorded
spp.	Multiple species within a specific genus
UR	The number of use records
UV	Use value
